# The Intermittent of Calc-carb

**Published:** 1893-06

**Authors:** A. McNeil

**Affiliations:** San Francisco, Cal.


### THE INTERMITTENT OF CALCAREA-CARB.

A. McNeil, M. D., San Francisco, Cal.

I give the following description of an intermittent fever curable by Calcarea-carb. and other drugs from Bœnninghausen. I reproduce it the more cheerfully because it has been omitted from the *Guiding Symptoms.*

"Intermittent fever with swelling of the joints and glands, particularly those of the knees and lower jaw, almost every time was cured by Calcarea-carb., so that the metastatic complaints never occurred. These fevers were characterized thereby, that the chill and heat either rapidly alternated or were present at the same time; the chill being internal, the heat external, usually accompanied by palpitation of the heart and followed by profuse sweat, which had the disposition to persist a long time in convalescents, and it had the peculiarity that it occurred more freely on motion in the cold open air and only in the first sleep in bed (Ars.), and not in the morning.

The weakness and prostration during paroxysm which Calc-carb. has, is on no account to be considered as a criterion, for there are many remedies corresponding to such cases. For example, we have seen such prostrating intermittents in which violent headache, interrupted by sopor with dreams (Arsenic). Others with violent rheumatic pains in the limbs, with insatiable thirst and frequent discharge of urine (Lycopodium). And still others in which distress in the stomach, with vomiting everything taken into it, followed by rush of blood to the head and difficulty of breathing (Ferrum-met.).

Key-note, Sulphur; cold sweat on the neck.

Sepia: sweat more profuse after than during exercise.

Opium: collapse with no natural reaction of the vital powers; he lies as if dead, the entire body being cold and stiff; face bluish, bloated, and covered with sweat.

Stramonium: During the day weeping prevails, while at night spasmodic laughter predominates.

Ipecac., Hæmaturia of (neat) cattle when first going to pasture and toward autumn.

Colchicum : For cattle that have eaten clover and have become dangerous by reason of the flatulent colic thus engendered.

Bismuth : Hiccough after vomiting.

Cuprum : Vomiting after hiccough.

Lach. : After the fever, hiccough and vomiting simultaneously.

Phosph-ac. : Sullen, indifferent—taciturn and sad disposition.

Kali-carb. : Vomiting of bluish colored blood.

Hepar : Pain in throat, ameliorated by talking.

Bell. : Inflammation of the brain caused by sunstroke, when very recent.

Camphor : Sunburn on face and hands—(internally in a high potency).

